# First record of Forcipomyia (Forcipomyia) pinamarensis Spinelli, 1983 (Diptera, Ceratopogonidae, Forcipomyiinae, Forcipomyiini) from Colombia

**DOI:** 10.3897/BDJ.14.e186552

**Published:** 2026-05-12

**Authors:** Jean Gamboa, Juan Hoyos, Eidy Martínez-Viuche, Ervin Humprey Duran-Bautista, Francisco Serna

**Affiliations:** 1 Universidad de la Amazonia, Laboratorio de Entomología Universidad de la Amazonia – LEUA, Grupo de Investigación en Entomología Universidad de la Amazonia – GIEUA, Florencia, Colombia Universidad de la Amazonia, Laboratorio de Entomología Universidad de la Amazonia – LEUA, Grupo de Investigación en Entomología Universidad de la Amazonia – GIEUA Florencia Colombia; 2 Universidad de la Amazonia, Laboratorio de Ecología del Suelo, Grupo de Investigación en Agroecosistemas y Conservación en Bosques Amazónicos – GAIA, Florencia, Colombia Universidad de la Amazonia, Laboratorio de Ecología del Suelo, Grupo de Investigación en Agroecosistemas y Conservación en Bosques Amazónicos – GAIA Florencia Colombia; 3 Universidad Nacional de Colombia, Sede Bogotá, Museo Entomológico UNAB, Grupo de Investigación en Sistemática de Insectos Agronomía - SIA, Bogotá, Colombia Universidad Nacional de Colombia, Sede Bogotá, Museo Entomológico UNAB, Grupo de Investigación en Sistemática de Insectos Agronomía - SIA Bogotá Colombia

**Keywords:** agroecosystem, Amazonia, biting midges, cacao, distribution, pollination, ecology

## Abstract

**Background:**

Forcipomyia (Forcipomyia) pinamarensis Spinelli, 1983 is recorded for the first time from Colombia in a cacao-producing agroecosystem in the Municipality of El Doncello, Department of Caquetá.

**New information:**

Detailed morphological characters and photographic documentation for the species Forcipomyia (Forcipomyia) pinamarensis Spinelli, 1983 are provided. Two specimens (one female and one male) of the species were recorded in an agroforestry system of *Theobroma cacao* L. (Malvaceae) - Cacao and Musa × paradisiaca (Musaceae) - Plantain located in the micro-basin of the Doncello River in the Napo Province of Colombia.

## Introduction

Genus *Forcipomyia* Meigen, 1818 (Diptera, Ceratopogonidae, Forcipomyiinae, Forcipomyiini) has approximately 38 subgenera and aproximately 1,200 species worldwide (Borkent and Dominiak 2020, Borkent et al. 2025). In the Neotropical Region, the genus is remarkably diverse with approximately 235 recorded species (Santarém and Felippe-Bauer 2024, Correa et al. 2025). The diagnoses of the biting midges of the genus *Forcipomyia* and subgenus Forcipomyia Meigen, 1818 are included in de Meillon and Wirth (1991)and Alwin-Kownacka et al. (2016), respectively.

Biting midges of *Forcipomyia* have been associated with the pollination of *Theobroma cacao* L. (Malvaceae) (Saunders 1959,Soria and Wirth 1979,Wirth 1982, Wirth 1991, O’Doherty and Zoll 2012). However, interactions amongst the cacao tree - pollinator *Forcipomyia* spp. are relatively poorly understood, despite the global importance of this crop, which is widely cultivated across the Tropics (Arnold et al. 2019). Pollination rates in cacao are often low and yields are poor in many countries (Groeneveld et al. 2010, Forbes and Northfield 2017, Arnold et al. 2019).

In Colombia, 50 species of *Forcipomyia* are recorded from the Departments of Antioquia, Caquetá, Cauca, Chocó, Meta, Risaralda and Valle del Cauca (Spinelli and Wolff 2016). Amongst those species, the subgenus Forcipomyia is represented by the species Forcipomyia (Forcipomyia) argenteola Macfie, 1939; Forcipomyia (Forcipomyia) calatheae Wirth, 1982; Forcipomyia (Forcipomyia) genualis (Loew, 1866); Forcipomyia (Forcipomyia) harpegonata Wirth & Soria, 1975; and Forcipomyia (Forcipomyia) catarinensis Marino & Spinelli, 2002 with records in Antioquia and Valle del Cauca (Spinelli and Wolff 2016).

In Caquetá, Forcipomyia (Caloforcipomyia) hatoensis Utmar & Wirth, 1976 and Forcipomyia (Microhelea) insignipalpis Macfie, 1949 are the only recorded species (Spinelli and Wolff 2016). This work constitutes the first record of the subgenus Forcipomyia from the Department of Caquetá in Colombia.

The species Forcipomyia (Forcipomyia) pinamarensis Spinelli, 1983 was previously recorded in six countries of the Neotropical Region (Spinelli 1983, Wirth 1991, Cazorla et al. 2018, Borkent and Dominiak 2020, Spinelli et al. 2023). This study aims to report the first record of F. (F.) pinamarensis from Colombia, expanding its known distribution and providing morphological data to facilitate its identification in cacao agroecosystems.

## Materials and methods

In 2024, sampling of Ceratopogonidae was carried out in 52 farms, located in the micro-basin of the Doncello River in the Municipality of El Doncello, Department of Caquetá, in Colombia. A 400g sample of decomposing cacao fruit husks at the El Paraíso farm was collected. The sample was placed in a plastic bag and transported to Laboratorio de Entomología Universidad de la Amazonia (LEUA), in the city of Florencia (Caquetá, Colombia).

The sample was placed in an emergency chamber consisting of a plastic box [28 cm (long) x 18 cm (wide) x 11 cm (high)] with a lid that had a central hole 10 cm in diameter covered with a fine-mesh wire grid to allow air passage, light entry and moisture exchange. A 250 ml plastic bottle with a fine-mesh white fabric base was placed on top of the mesh and next to it, a 25 ml plastic bottle containing ethyl alcohol (96%) was connected, in which the emerging adult specimens were collected.

The curatorship of the specimens was performed using a chemical process [96% alcohol (1 h), 50% alcohol + 50% amyl acetate (1 h), 100% amyl acetate (2 h), 100% amyl acetate (1 h) and then drying at room temperature] and point-mounting the specimens following the protocols established in the LEUA insect collection (Pérez-Benavides et al. 2023). The identification of the specimens was possible through specialised literature (de Meillon and Wirth 1991, Wirth 1991, Borkent and Spinelli 2007, Borkent 2024).

Colour images and measurements of the specimens included in this work were taken using a LEICA M205A stereomicroscope. Images SEM were taken using a HITACHI TM4000 Plus II environmental scanning electron microscope from specimens on point-mounting. A distribution map of the species was created using QGIS v. 3.26.2. The figures were prepared using Photoshop 2023 V. 24.0.

## Taxon treatments

### Forcipomyia (Forcipomyia) pinamarensis

Spinelli, 1983

2EC30291-69BE-5866-A0D9-2A3C062350B2

#### Materials

- **Type status:** Other material. **Occurrence:** occurrenceID: 6BCD3742-9ACE-5911-99EC-730D7786A5BC; **Taxon:** scientificNameID: Forcipomyia (Forcipomyia) pinamarensis Spinelli, 1983; higherClassification: Invertebrate; kingdom: Animalia; phylum: Arthropoda; class: Insecta; order: Diptera; family: Ceratopogonidae; genus: Forcipomyia; subgenus: Forcipomyia; specificEpithet: *pinamarensis*; scientificNameAuthorship: Spinelli, 1983; **Location:** continent: America; country: Colombia; stateProvince: Caquetá; municipality: El Doncello; locality: Vda. Los Laureles, Fca. El Paraíso; locationRemarks: COLOMBIA — CAQUETÁ • El Doncello, Vda. Los Laureles, Fca. El Paraíso; 01°41′49″N, 75°17′48″W; 609 m alt.; 08.IV.2024; J. Hoyos leg.; manual collection from an emergency chamber that contained larvae on decomposing cacao fruit shells collected in an agroforestry system of Theobroma cacao L. (Malvaceae) – Cacao and Musa x paradisiaca L. (Musaceae) – Plantain; verbatimCoordinateSystem: 01°41′49″N, 75°17′48″W; **Event:** eventID: LEUA-22468/22469; samplingProtocol: manual collection from an emergency chamber; eventDate: 4/8/2024; habitat: agroecosystem

#### Description

**Figure 1.**
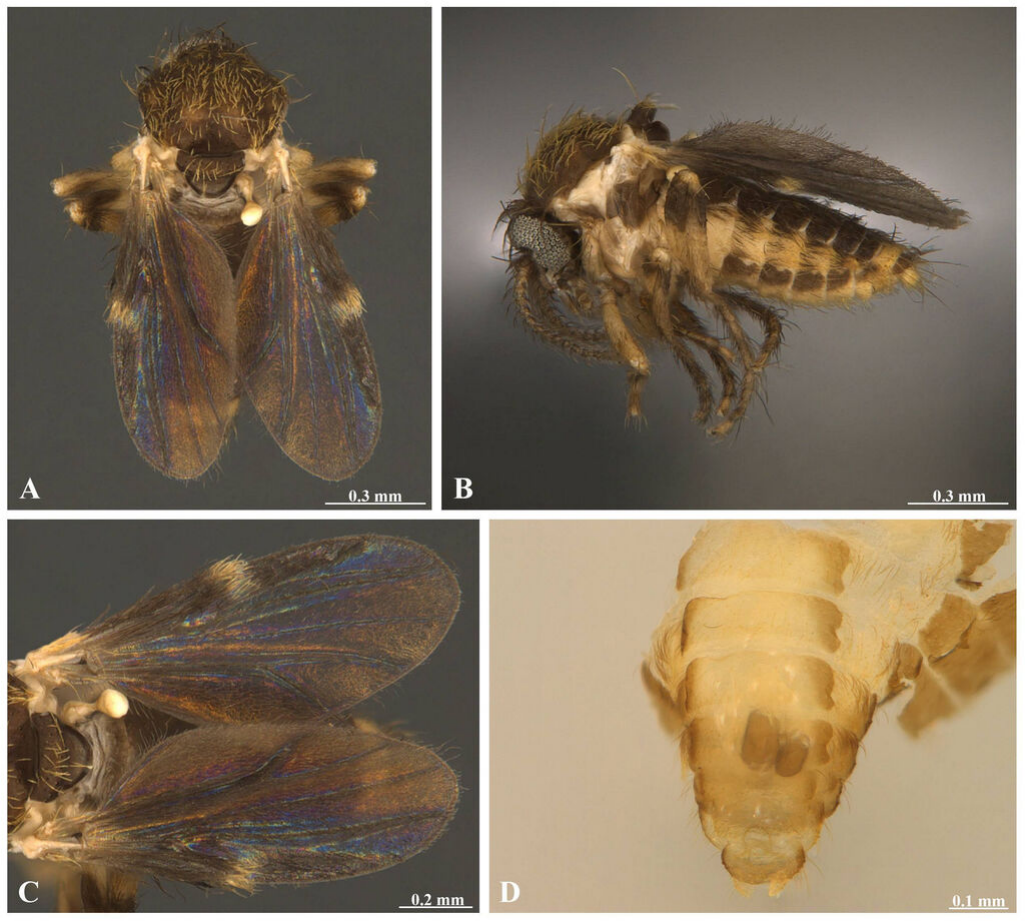
Forcipomyia (Forcipomyia) pinamarensis (Spinelli, 1983), female. **A–B** Habitus: **A** Dorsal view; **B** Lateral view; **C** Wing, dorsal view; **D** Spermathecae, ventral view.

**Identification.** Features agree with the re-description of F. (F.) pinamarensis carried out by Wirth (1991): **Adult female.** Length 1.60–1.70 mm. Head (anterior view) pale brown; dark ring around bases of antenna; palpus, segments 1 to 5 0.10x, 0.12x, 0.41x, 0.23x and 0.14x as long as the palpus total length, brown with light bands at distal articulations, segment 3, proximal one-third swollen, distal neck tapering gradually; antenna, 0.53–0.60 mm, segments 1 to 14 0.10x, 0.08x, 0.07x, 0.07x, 0.07x, 0.06x, 0.06x, 0.06x, 0.06x, 0.06x, 0.06x, 0.06x, 0.07x and 0.11x as long as the antenna total length, scape light brown, flagellum light brown (basally) to brown (apically) (Fig. 1 and Fig. 3). Thorax (dorsal view) dark brown with at least four light brown spots; humeral angles white; scutellum brown to dark brown around margins; postscutellum dark brown; pleura light brown on all the usual sclerites; a very small sclerite in front of the base of the wing dark brown; wing, length 1.12–2.11 mm, width 0.45–0.46 mm, translucent dark brown, branches of radius dividing to form distinct cell, costa apex at middle of wing, fork of Cu directly below, densely covered with dark hairs; haltere whitish; hind tibia with hastate spines, hind tarsal segments, 0.33x, 0.27x, 0.16, 0.13x and 0.11x as long as the tarsus total length (Fig. 1 andFig. 3). Abdomen, tergites uniformly brown, except 8 which is orange and brown; sternites 3-7 orange-brown with dark brown lateral spots, 8 orange and brown; cerci orange and brown; tergites and pleural membrane clothed with narrow black-striated hairs, pale simple hairs on venter; spermathecae two, 0.090 by 0.056 mm, subspherical (Fig. 1).

**Remarks.** The Colombian specimen has palpus segment 3 with a proximal circular hole of diameter 0.013 mm (Fig. 1 and Fig. 3). Antennal segment 1 copiform, 2 to 13 flask-shaped distally elongated, 14 flask-shaped slightly widened distally (Fig. 1 and Fig. 3). Thorax with abundant setae of different sizes; scutellum with long setae posterioriorly; haltere copiform, proximally 0.21x as wide as total length, distally 0.49x as wide as total length, glabrous (Fig. 1 and Fig. 3). Wing densely covered with dark microtrichia, except for cell r2 with distal whitish microtrichia (Fig. 1 and Fig. 3). Fore- and middle legs, coxa, trochanter, femur and tibia white and pale brown, fore- tarsus white and middle tarsus brown; hind leg, coxa and trochanter white, femur white (proximally) and brown (distally), tibia and tarsus brown (Fig. 1 and Fig. 3). Fore- and middle tibiae with hastate spines (Fig. 3).

**Figure 2.**
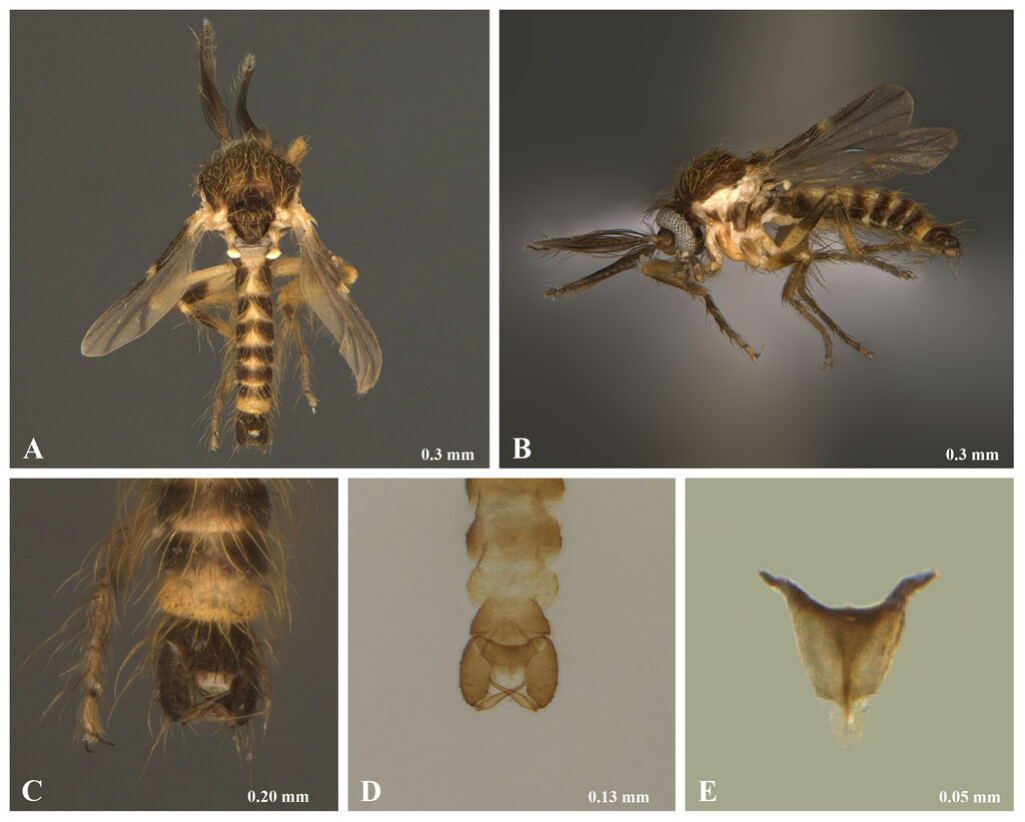
Forcipomyia (Forcipomyia) pinamarensis (Spinelli, 1983), male. **A–B** Habitus: **A** Dorsal view; **B** Lateral view; **C** Abdomen, posterior part; **D–E** Genitalia: **D** Dorsal view; **E** Aedeagus.

**Figure 3.**
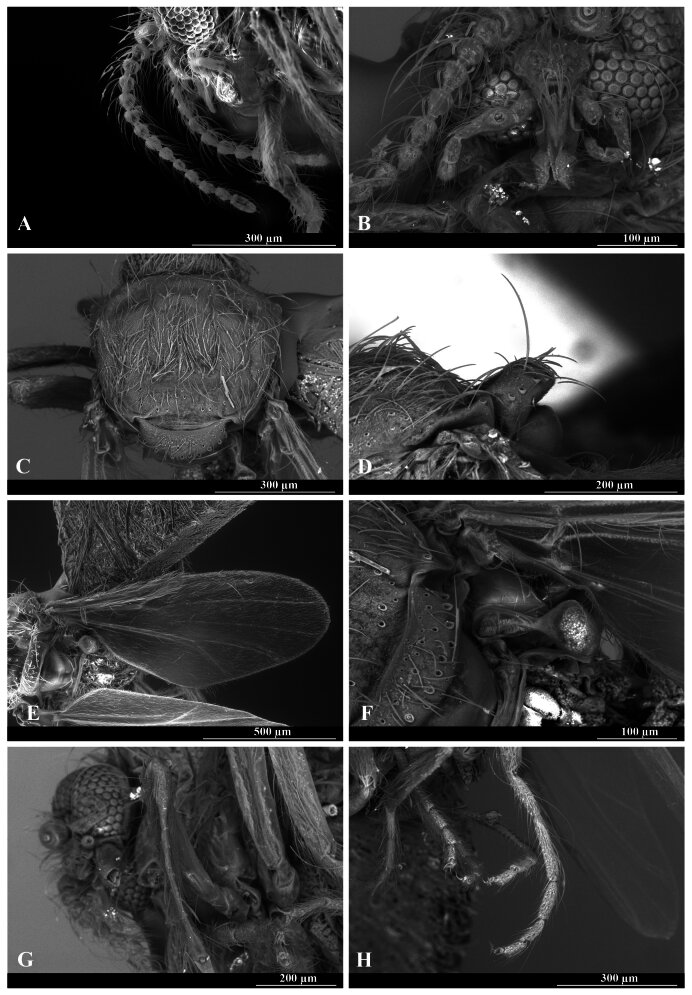
Forcipomyia (Forcipomyia) pinamarensis (Spinelli, 1983), female. **A** Antenna, lateral view; **B** Palpus, anterodorsal view; **C** Thorax, dorsal view; **D** Scutellum, lateral view; **E** Wing, dorsal view; **F** Halter, lateral view; **G** Hastate spines of fore tibia, lateral view; **H** Hind tarsi, lateral view.

**Male.** Palp with apical segment longer than in female; segments 1 to 5, 0.10x, 0.12x, 0.40x, 0.21x and 0.17x as long as the palpus total length (Fig. 2). Hypopygium: aedeagus long, rounded apically, hairy, without lateral carinae (Fig. 2). Parameres joined in middle, a slender pair of bowed stylets extend backwards beyond aedeagus apex (Fig. 2). Side pieces of forceps long, slightly tapered, with distinct brush of fine hairs on inner side near anterior end (Fig. 2). The Colombian specimen has a colouration pattern similar to the female (Fig. 1 and Fig. 2).

#### Distribution

Argentina, Brazil, Costa Rica, Panama, Uruguay and Venezuela (Spinelli 1983, Wirth 1991, Cazorla et al. 2018, Borkent and Dominiak 2020, Spinelli et al. 2023).

#### Notes

**Figure 4.**
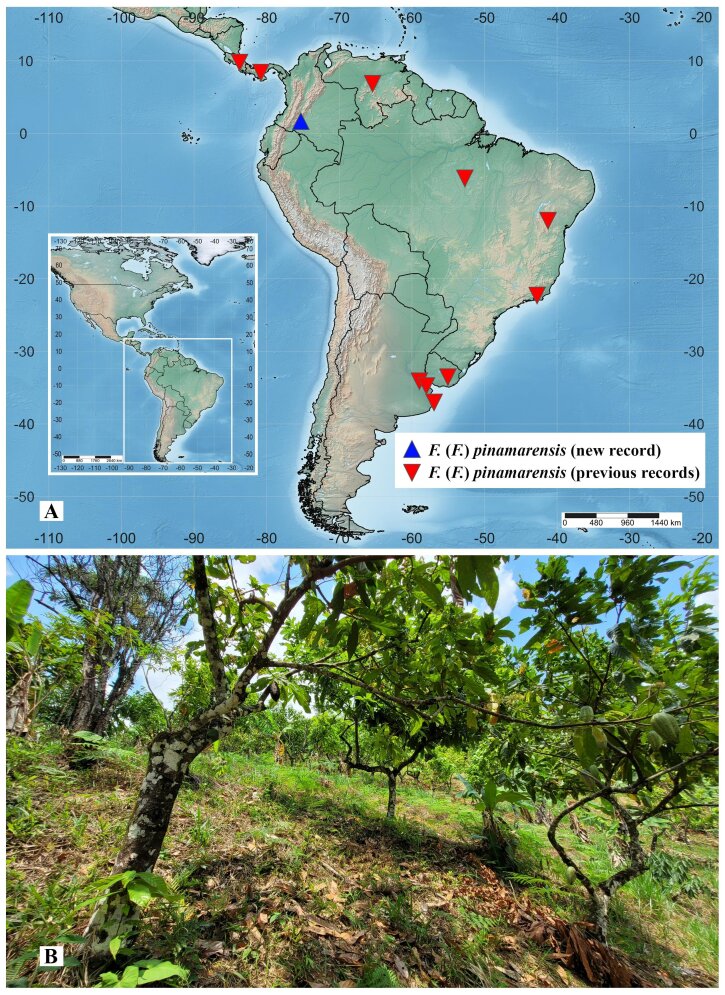
Ecological aspects of Forcipomyia (Forcipomyia) pinamarensis (Spinelli, 1983). **A** Geographical distribution; **B** Habitat.

The current known distribution of the species is Argentina, Brazil, Colombia, Costa Rica, Panama, Uruguay and Venezuela (Fig. 4). All of those countries are located within the Neotropical Region (Morrone 2001).

The collection locality of the specimens examined in this work corresponds to the Napo Province, in the Amazonia subregion of the Neotropical Region (Morrone 2001). The area is predominantly covered by humid forests, with an an extensive system of meandering rivers that create habitat mosaics (Dinerstein et al. 1995).

## Discussion

Natural populations of *T. cacao* are distributed in existing forests from the Amazon Basin of South America to southern Mexico, with trees growing under the understorey shade in those rainforests (Whitkus et al. 1998, Schroth et al. 2004). Small farmers cultivate cacao in geographic areas that correspond to the Tropical Rainforest in various countries in the Americas (Somarriba and Lachenaud 2013). In Colombia, there are 65,341 rural family communities producing cacao, in a total area of 256,325 hectares (ha) (MADR 2021, UPRA 2023). In the Department of Caquetá, cacao plantations are managed employing traditional technologies, with an average dry cacao bean yield of 0.4 t.ha^-1^.yr^-1^ (FEDECACAO 2021, UPRA 2023).

In the micro-basin of the Rio Doncello River, farmers establish cacao trees in association with species, such as *Cariniana pyriformis* Miers (Lecythidaceae), *Hevea brasiliensis* (Willd. ex A. Juss.) Müll. Arg. (Euphorbiaceae), *Theobroma bicolor* Bonpl. (Malvaceae), *Theobroma grandiflorum* (Willd. ex Spreng.) K. Schum. (Malvaceae), *Cecropia* sp. (Urticaceae), *Inga spuria* Humb. & Bonpl. Ex Willd. (Fabaceae), *Musa* x *paradisiaca* L. (Musaceae), *Manihot esculenta* Crantz (Euphorbiaceae), *Zea mays* L. (Poaceae) and *Coffea arabica* L. (Rubiaceae), stablishing agroforestry systems. In the region, according to Suárez et al. (2018), at least four types of traditional cacao agroforestry systems are recognised: 1) complex diversified multi-strata; 2) low diversity with regular trees; 3) low diversity with grouped trees and 4) high density of Musaceae. According with Suárez et al. (2018), the cacao agroecosystems of the farm El Paraíso, where F. (F.) pinamarensis was found, corresponds to typology 4 (Fig. 4). Diversified cacao agroecosystems provide habitat and food for a greater richness of pollinating insects of cacao trees.

In these cacao agroforestry systems, a high diversity of Ceratopogonidae was found, corresponding to 14 genera of the subfamilies Forcipomyiinae (*Atrichopogon* Kieffer, 1906; *Dasyhelea* Kieffer, 1911; and *Forcipomyia* Meigen, 1818) and Ceratopogoninae (*Amerohelea* Grogan & Wirth, 1981; *Brachypogon* Kieffer, 1899; *Clastrieromyia* Spinelli & Grogan, 1985; *Clinohelea* Kieffer, 1917; *Culicoides* Latreille, 1809; *Downeshelea* Wirth & Grogan, 1988; *Echinohelea* Macfie, 1940; *Mallochohelea* Wirth, 1962; *Monohelea* Kieffer, 1917; *Stilobezzia* Kieffer, 1911; and *Bezzia* Kieffer, 1899). Several species of these genera are recognised as pollinators of *T. cacao*. For example, F. (F.) pinamarensis is recognised as a pollinator of cacao trees (Sánchez et al. 2001, Alvarado et al. 2018).

A major factor contributing to low cacao production is poor pollination. During the development of this work, we found that many traditional cacao plantation management practices destroy the habitat and food sources of cacao-pollinating mosquitoes. For example, the larvae and pupae of F. (F.) pinamarensis inhabit sites where cacao fruit husks are piled up during decomposition, so it is incorrect to apply quicklime to those sites as a soil management technique for pH regulation. Another practice worth stressing is the implementation of cacao monoculture. Shade reduction decreases soil moisture, alters the conditions necessary for the decomposition of organic matter and disrupts the habitat of the immature stages of F. (F.) pinamarensis and other pollinating species.

This work records the third species of the genus *Forcipomyia* and the first of the subgenus Forcipomyia from the Department of Caquetá. However, the high diversity of *Forcipomyia* in this region remains largely unexplored. New studies focused on the description of new species and new records of species from Colombia will be carried out.

## Supplementary Material

XML Treatment for Forcipomyia (Forcipomyia) pinamarensis
